# The Validity and Reliability Characteristics of the M-BACK Questionnaire to Assess the Barriers, Attitudes, Confidence, and Knowledge of Mental Health Staff Regarding Metabolic Health of Mental Health Service Users

**DOI:** 10.3389/fpubh.2017.00321

**Published:** 2017-12-11

**Authors:** Andrew Watkins, Simon Rosenbaum, Philip B. Ward, Joanna Patching, Elizabeth Denney-Wilson, Jane Stein-Parbury

**Affiliations:** ^1^Keeping the Body in Mind Program, The Bondi Centre, South Eastern Sydney Local Health District, Sydney, NSW, Australia; ^2^Faculty of Health, University of Technology Sydney, Sydney, NSW, Australia; ^3^School of Psychiatry, University of New South Wales, Sydney, NSW, Australia; ^4^Schizophrenia Research Unit, Ingham Institute for Applied Medical Research, Liverpool, NSW, Australia; ^5^School of Nursing, University of Notre Dame, Sydney, NSW, Australia

**Keywords:** severe mental illness, physical health, education, training, evaluation tool, service delivery, health outcomes, metabolic syndrome

## Abstract

**Background:**

Addressing the burden of poor physical health and the subsequent gap in life expectancy experienced by people with mental illness is a major priority in mental health services. To equip mental health staff with the competence to deliver evidence-based interventions, targeted staff training regarding metabolic health is required. In order to evaluate the effectiveness of staff training regarding metabolic health, we aimed to develop a succinct measure to determine the barriers, attitudes, confidence, and knowledge of health practitioners through the development and test–retest reliability of the Metabolic-Barriers, Attitudes, Confidence, and Knowledge Questionnaire (M-BACK).

**Methods:**

The M-BACK questionnaire was developed to evaluate the impact of specialized training in metabolic health care for mental health nurses. Content of the M-BACK was developed from a literature review and refined by an expert review panel and validated *via* a piloting process. To determine the test–retest reliability of the M-BACK, 31 nursing students recruited from the University of Notre Dame, Sydney completed the questionnaire on two separate occasions, 7 days apart. Intraclass correlation coefficients (ICCs) were calculated for the total score, as well as each of the four domains.

**Results:**

Pilot testing was undertaken with a sample of 106 mental health nurses with a mean age 48.2, ranging from 24 to 63 years of age, who participated in six training courses. Questionnaire development resulted in a 16-item instrument, with each item is scored on a five-point Likert scale ranging from “strongly disagree” to “strongly agree.” Test–retest reliability of the M-BACK was completed by 30 of 31 nursing students recruited, ICCs ranged from 0.62 to 0.96.

**Conclusion:**

The M-BACK is a reliable measure of the key elements of practitioner perceptions of barriers, and their knowledge, attitudes, and confidence regarding metabolic monitoring in people with mental illness. It can be used to assess the effectiveness of interventions aimed at increasing uptake of metabolic monitoring, a key component of programs to reduce the life expectancy gap in people living with severe mental illness.

## Introduction

People with mental illness have poorer physical health outcomes in comparison to the general population. Severe mental illness (SMI), including schizophrenia and related psychotic disorders, bipolar disorder, and major depressive disorder, are associated with a reduced life expectancy of between 10 and 20 years (1, 2), primarily due to preventable cardiometabolic diseases. People with these disorders are more likely than the general population to meet criteria for metabolic syndrome (MetS) (3), a cluster of risk factors including abdominal obesity, dyslipidaemia, hypertension, and insulin resistance (4).

The Second Australian National Survey of Psychosis study reported that 60.8% of people who experienced psychosis had MetS, which is two to three times that of the general Australian population (5). People with a serious mental illness (SMI) have other risk factors including rates of smoking more than twice that of the general public, higher rates of obesity, and lower levels of physical activity than the general population (5–7). While the advent of second-generation antipsychotics has reduced some side effects of their first-generation predecessors, the metabolic profile of these medications has led to increasing rates of MetS in people prescribed these medications (8, 9).

Metabolic health disorders that commonly occur with second-generation antipsychotics have not been well managed by mental health services (5). There is a general lack of expertise, confidence, and practical experience in addressing physical health care among all mental health professionals (10, 11). As a result of diagnostic overshadowing, mental health nurses in particular have traditionally focused on addressing mental illness symptomatology, while the physical health care needs of service users have often been a lower priority (12).

Mental health nurses are well positioned to play a key role in providing metabolic health care to people who experience mental illness (13). While mental health nurses acknowledge that they have a role in addressing physical health needs (14), they may not have the skills or knowledge to identify and manage the metabolic complications experienced by service users, prompting recommendations for specific training in this area (15–17). Barriers to mental health professionals proactively tackling the high burden of poor physical health include: a lack of time, poor knowledge, and a lack of confidence regarding metabolic screening and the provision of interventions aimed at reducing metabolic risk (18, 19). However, there is evidence that brief training can lead to improvements in rate of metabolic monitoring in inpatient settings (20).

In addition to metabolic monitoring, there is increasing evidence demonstrating the effectiveness of lifestyle interventions for preventing and treating cardiometabolic disease among people with SMI (21–24) in line with exhortation to “*don’t just screen, intervene*” (25). Drawing on literature from the general population, the essential components of effective lifestyle interventions are well established (22, 26), including the need for a multidisciplinary approach incorporating physical activity and nutritional components along with health coaching. Evidence for the role of lifestyle interventions in reducing overall cardiometabolic risk and obesity among people with SMI has been described as overwhelming, with gaps in service delivery considered an “implementation gap,” as opposed to a “knowledge gap” (23).

Two clinical tools that measure the effectiveness of metabolic health care have been evaluated in mental health professionals. The first of these is the Physical Health Attitude Scale (PHASe) (27), a 28-item measure that assesses attitudes of mental health nurses toward physical health care of mental health service users. The PHASe tool has primarily been used to obtain a cross-sectional snapshot of the perceptions and practices of mental health nurses regarding the general physical health of service users (14, 28, 29). However, this tool does not measure knowledge change, which is a key training goal, and is not specifically focused on the area of metabolic health care. Stanton et al (30) recently developed a questionnaire [the Exercise in Mental Illness Questionnaire—Health Professionals Version (*EMIQ-HP*)] to investigate the knowledge, attitudes, beliefs, and behaviors of health professionals regarding exercise in the treatment of mental illness. The EMIQ-HP demonstrated excellent test–retest reliability. While valuable, its focus is limited to one modifiable risk factor, physical activity, and therefore is not ideal to assess changes regarding metabolic health education more broadly.

Physical health care incorporates a broad and diverse range of health specialties. While there are many domains of physical health care for people experiencing SMI that warrant careful attention (5), metabolic health is an area of particular concern (31). Specialized training for mental health nurses in metabolic health screening and interventions is therefore a priority. The ability to measure the effectiveness of this type of training for mental health nurses is critical.

The objectives of the study were to (1) create a succinct tool that could measure changes in perceived barriers, attitudes, confidence, and knowledge of nurses related to metabolic health care in mental health service users, (2) ensure that tool contain valid content, and (3) the instrument has reliability as to be sensitive to change to determine the effectiveness of training, education, or other initiative to improve the delivery of metabolic health care by mental health clinicians.

## Materials and Methods

The development of the M-BACK commenced with a review of the literature to identify the training needs, barriers, and enablers associated with metabolic monitoring and the delivery of lifestyle interventions by mental health nurses, psychiatrists, and other allied mental health staff. Findings from that review indicated that the attitudes of clinical staff toward providing metabolic health care in mental health settings was integral to the successful delivery of such services (14, 15, 19, 27, 28). Another common finding was a low level of confidence among mental health staff in carrying out recommended metabolic health monitoring and delivering interventions (15, 16, 18, 32), in addition to a lack of knowledge in this area (15, 33, 34). Other consistent barriers to staff delivering metabolic care were workload, concerns regarding medication adherence, perceived apathy on the part of a service user or a sense of hopelessness from a staff member (15, 16, 18, 19, 27, 28, 33–35).

### Content Validation

Twenty questions were developed based upon four themes that were identified in the literature: barriers, attitudes, confidence, and knowledge of metabolic health care. The questionnaire was then reviewed by a panel of seven people with specialist expertise in the delivery of metabolic health care to mental health service users including clinicians, educators and researchers. Experts were chosen based upon criteria requiring them to be published experts in the field of metabolic health care of mental health service users along with clinical and/or teaching experience in this area. There was also a desire for the panel to be representative of the major disciplines in mental health care. Therefore, the panel included members from mental health nursing, psychiatry and psychology. In addition, the panel featured an exercise physiologist and dietitian that work in mental health care. Each expert reviewer was sent a copy of the questionnaire and an explanation as to the purpose of the instrument and asked to comment on the applicability, clarity, and simplicity of each item. Reviewers were also encouraged to provide comment on the overall layout and design of the questionnaire. After written feedback, six of the expert panel met to cull and refine the questions. At the conclusion of the expert consensus review 16 Likert-type questions were produced across four domain areas: barriers to delivering metabolic health care, attitudes toward metabolic health care, confidence in delivering metabolic health care, and knowledge of metabolic health care.

### Pilot Testing

The 16-item M-BACK tool was pilot tested in 2013 on participants completing a training course, in order to ascertain their understanding of the statements employed in the questionnaire. The training consisted of a 2-day workshop on metabolic health care for people with a mental illness and delivered at the Australian College of Nursing. The training was designed and delivered by lead author (Andrew Watkins) with the aim of improving participants’ knowledge and confidence in providing metabolic health care screening and interventions. Training included both educational and practical elements on topics such as: how to screen for metabolic health (e.g., taking blood pressure, waist circumference), nutritional interventions (e.g., conducting a basic dietetic assessment and providing nutritional advice), exercise interventions (e.g., assessing physical activity levels and prescribing exercise), and pharmacological interventions (e.g., medications that can be used to reduce metabolic risk factors).

The pilot testing was undertaken with a sample of 106 mental health nurses with a mean age 48.2, ranging from 24 to 63 years of age over six separate courses. Participants undertook pre and post testing of the M-BACK questionnaire. There was space provided below each question and instructions provided for participants to make comments if they found the question confusing or ambiguous. Feedback from participants was circulated to the expert panel, who were given 2 weeks to provide comment. Expert group comments were collated by lead author (Andrew Watkins). The expert panel met on two occasions during the piloting phase and made refinements to several of the questions to remove any identified ambiguity that had been identified in the pretesting phase. The refined questions were reviewed at two subsequent meetings, in order to reach agreement on the question content.

The penultimate version that was then piloted with the last cohort of workshop of 19 participants who did not offer any negative feedback in relation to confusion or ambiguity of the questions. The final version incorporated numeric coding (responses included numbers) to allow for ease of scoring and facilitate statistical analysis. This version achieved approval by all authors and the expert panel.

### Instrument Overview

The M-BACK (see Image S1 in Supplementary Material) is a 16-item instrument separated into four domains: knowledge, confidence, attitudes, and practice barriers in relation to metabolic health. Each item is a five-point Likert type scale ranging from Strongly Disagree (scoring 1) to Strongly Agree (scoring 5). Each domain is composed of four items and scored out of 20, with a minimum score of four and a maximum score of 20. Total score for the questionnaire is 80, with a minimum of 16 (see Image S2 in Supplementary Material).

### Domain 1: Barriers

Items 1–4 address barriers to metabolic screening and intervention, including, workload, service user interest, conflict with mental health goals, and inability to effect change. These questions are negatively posed with the scoring reversed. Possible scores for this domain range from 4 to 20.

### Domain 2: Attitudes

Items 5–8 investigate attitudes, including toward metabolic monitoring, the provision of smoking cessation advice, physical activity, and nutritional intake. Possible scores for this domain range from 4 to 20.

### Domain 3: Confidence

Items 9–12 assess the confidence of respondents in providing interventions to prevent or treat metabolic health including smoking cessation, physical activity and nutritional interventions. Possible scores for this domain range from 4 to 20.

### Domain 4: Knowledge

Items 13 through 16 assess knowledge of metabolic health, screening, interpreting pathology reports, and understanding of the metabolic side effects of neuroleptic medication. Possible scores for this domain range from 4 to 20.

### Instrument Reliability

The M-BACK questionnaire is intended to assess change over time and to detect change in the items following education or training. Therefore, test–retest reliability to assess the stability of a measure over time was of particular importance for the M-BACK. The test–retest reliability for each question was determined utilizing intraclass correlation coefficients (ICCs) using a two-factor mixed effects model (36). ICC was chosen as the statistical method under the presumption that a Likert-type scale scored items are generally considered as a continuous variable and ICC is the preferred method of assessing test–retest data under these conditions (37). Using ICC will determine the proportion of total variance that occurs between time points (36).

To determine the required sample size a method devised by Walter et al. was utilized to calculate the number of subjects required for a reliability study being measured by ICC (38). This method is based on a functional approximation to earlier exact results and has shown excellent agreement with the exact results. Based on this method, the sample size determined for the 16-item scale was 29.

Thirty-two final year undergraduate nursing students from the University of Notre Dame in Sydney who had elected mental health as a specialty were invited to complete the questionnaire. There were no other exclusion criteria. Students were offered a verbal invitation to complete the questionnaire at the conclusion of a mental health lecture on therapeutic relationships. Student participants were informed of the research nature of completing the M-BACK, informed that participation was completely voluntary and there were no consequences for not participating. All participants completed a signed consent form. All 32 students completed the questionnaire on the first occasion with one person missing for the repeat questionnaire, due to illness. The sample of 31 who completed the questionnaire on the two occasions, 7 days apart had a mean age 23.9 ± 6.6 years and a majority were female (61%).

All survey data were analyzed using SPSS version 23. The data were reviewed to ensure completeness. The person who was missing for the repeated questionnaire was excluded from the analysis. ICC’s and 95% confidence intervals (Cis) were calculated for the total score, as well as each of the four domains (barriers, attitudes, confidence, and knowledge). ICCs were calculated and interpreted based on Landis and Koch, with ICCs of below 0.4 (poor to fair), 0.41–0.6 (moderate), 0.610.80 (excellent), and 0.81–1 (almost perfect) (39). The questionnaire was analyzed for individual items in addition to the four domain areas.

### Ethical Considerations

Ethical approval was granted for the study by University of Technology Sydney Human Research Ethics Committee (HREC 2013000749). The research was deemed by this ethics committee to be of negligible risk and not requiring of formal debriefing strategies for participants. Anonymity was assured by keeping demographic data separate from the completed questionnaires.

## Results

### Content Validity

The expert panel met to discuss the M-BACK tool (with the exception of overseas participants), followed by several rounds of email correspondence. Comments received from the expert panel were incorporated into the M-BACK instrument. The expert panel contributed to further development of the content in the “attitudes” and “knowledge” sections, and wording in the “confidence” questions. Two reviewers noted that the term “consumer” needed to be used consistently rather than utilizing “client.” A number of items were reworded to make them more specific regarding a nurse’s role. For example, the original item; “Encouraging consumers to increase their level of physical activity is an important part of my role,” was reworded to; “Encouraging consumers to increase their level of physical activity is an important part of my role as a mental health nurse.” Four questions from the original literature review were culled following feedback from the expert panel as they were deemed to be redundant after all the questions were refined.

Pilot testing of the M-BACK tool allowed for feedback from course participants regarding their understanding of the statements employed in the questionnaire. The expert panel met on two occasions during the piloting phase and made refinements to several of the questions to remove ambiguity. The barriers section in particular was reworded in order to clarify that the questions were negatively framed. For example, the original item “There is no point to metabolic health screening for mental health consumers,” was reworded to “Screening for metabolic syndrome and physical health interventions are pointless as poor physical health outcomes are unavoidable.” Similarly, “I’m too busy to do health promotion work with clients” was adjusted to “My workload prevents me doing any health promotion activities with consumers.”

### Instrument Reliability

Thirty-one nursing students, mean age 23.9 ± 6.6 years (61% female) participated and completed the M-BACK on two occasions, 7 days apart. ICC correlations for individual items ranged from 0.62 to 0.96 (see Table 1).

**Table 1:** Intraclass correlation coefficient items.

Item subscales	Intraclass correlation coefficient	95% CI
**Barrier**
*My workload prevents me doing any health promotion activities with clients*	0.86	0.70–0.93
*Consumers with a serious mental illness are not interested in improving their physical health*	0.92	0.82–0.96
*Informing clients about the possible effects of medications may have on their mental health will increase non-adherence*	0.91	0.81–0.96
*Screening for metabolic syndrome and physical health interventions are pointless as poor physical health outcomes are unavoidable*	0.74	0.46–0.87

**Attitudes**
*Metabolic health screening is an important part of my role as a mental health clinician*	0.93	0.86–0.97
*Giving smoking cessation advice is an important part of my role as a mental health clinician*	0.81	0.60–0.91
*Encouraging consumers to increase their level of physical activity is an important part of my role as a mental health clinician*	0.78	0.54–0.89
*Discussing nutritional intake is an important part of my role as a mental health clinician*	0.82	0.63–0.91

**Confidence**
*I am confident in my ability to screen for metabolic syndrome*	0.92	0.83–0.96
*I am confident in providing smoking cessation advice to consumers*	0.87	0.73–0.94
*I am confident in prescribing exercise interventions to prevent/treat metabolic syndrome*	0.82	0.62–0.91
*I am confident in using dietary interventions to prevent/treat metabolic syndrome in consumers*	0.73	0.44–0.87

**Knowledge**
*I have a good knowledge of metabolic syndrome*	0.87	0.74–0.94
*I understand how to screen for metabolic syndrome*	0.96	0.91–0.98
*I understand how to read pathology reports for lipids and glucose results*	0.84	0.66–0.92
*I understand the metabolic side-effect profiles of different neuroleptic medication*	0.62	0.20–0.82

### Total Score

A high degree of reliability was found between the total M-BACK scores at both time points. The single measure ICC was considered excellent, 0.87 (95% CI 0.75–0.94). The mean between day variation was 0.54 ± 5.3.

### Barriers

The ICCs for the barrier items ranged from 0.74 (Item 4 “Screening for metabolic syndrome and physical health interventions are pointless as poor physical health outcomes are unavoidable”) to 0.92 (Item 2 “Consumers with a serious mental illness are not interested in improving their physical health”). The mean ICC was 0.71 (95% CI 0.39–0.86).

### Attitudes

The ICCs for the attitude items ranged from 0.78 (Item 3 “Encouraging consumers to increase their level of physical activity is an important part of my role as a mental health clinician”) to 0.93 (Item 1 “Metabolic health screening is an important part of my role as a mental health clinician”). The mean ICC was 0.80 (95% CI 0.58–0.90).

### Confidence

The ICCs for the confidence items ranged from 0.73 (Item 4 “I am confident in using dietary interventions to prevent/treat metabolic syndrome in consumers”) to 0.92 (Item 1 “I am confident in my ability to screen for metabolic syndrome”). The mean ICC was 0.88 (95% CI 0.75–0.94).

### Knowledge

The ICCs for the knowledge items ranged from 0.62 (Item 4 “I understand the metabolic side-effect profiles of different neuroleptic medication”) to 0.96 (Item 2 “I understand how to screen for metabolic syndrome”). The mean ICC was 0.90 (95% CI 0.80–0.96).

## Discussion

The article describes the development, content validity, and test–retest reliability of a novel tool, that assesses the attitudes, confidence, knowledge, of mental health nurses in providing metabolic health care to mental health consumers and the barriers perceived in implementing this care.

The fact that metabolic health is a primary driver of premature mortality among people with SMI is well established (25, 40–43). Unfortunately, mental health nurses often feel that they are ill-prepared to be able to screen and intervene for metabolic health and feel that they require training to rectify this (15, 32). The M-BACK may be of assistance in determining the reasons why metabolic screening and interventions are not occurring in clinical practice, so that training and education can be designed in a targeted way.

Despite recognition of the importance of metabolic health training there is a lack of published information on physical health training for mental health nurses and minimal information on its effectiveness (17, 44). This has led to repeated calls in the nursing literature for education and training in physical health to be provided for mental health nurses and for these educational programs to be evaluated (14–16, 32, 44). This M-BACK tool can be employed to meet the identified gap in measuring the effectiveness of training and education for mental health nurses and other initiatives of mental health services to address metabolic health care. It also enables evaluation of the effectiveness of education regarding metabolic health care through pre- and posttraining testing. In a similar way the questionnaire can be used to determine effectiveness of staff-based interventions for mental health professionals.

Content validation of the M-BACK survey was achieved by expert consensus. It enabled the incorporation of the views of researchers, educators and clinicians working in the field to develop a tool that captures pertinent data and facilitates detailed analysis. The M-BACK tool is appropriate to assess current knowledge regarding metabolic health in service users with SMI. Nonetheless, revisions of the M-BACK tool may be required as new knowledge is gained. In addition, the current tool is intended to assess the views of mental health nurses; a version adapted for completion by service users and/or carers would be a valuable addition.

This study also examined the test–retest reliability of the M-BACK. The questionnaire demonstrated acceptable test–retest reliability, with an ICC of 0.87 for the total M-BACK score ranging between 0.61 and 1 for each item.

Test–retest reliability analysis will rarely achieve perfect results (45). Within the domains, the greatest variance was found within the knowledge domain and least variance within the attitudes domain. Item 4 of the knowledge domain (*I understand the metabolic side-effect profiles of different neuroleptic medication*) had the lowest ICC of any item throughout the questionnaire, although this was still classified as excellent according to the criteria of Landis and Koch (39). Possible explanations for why some items in a questionnaire may have greater variability using include changed knowledge or awareness of a participant, perhaps even prompted be having completed the questionnaire previously (45). Given the acceptable test–retest results, it was determined that no changes to questions were required.

### Limitations

This validation study of the M-BACK questionnaire is not without limitations. A narrow definition of SMI was utilized in order to focus on service users where the greatest metabolic health risk exists, reflecting the use of antipsychotics in this population. Similarly, we used mental health nurses as the targeted clinicians for this tool, as nurses tend to have the most face-to-face contact with service users. Instrument reliability was examined in nursing students during the final year of their undergraduate training. Although students were not the primary targets of the M-BACK tool, it is unlikely that utilizing students impacted test–retest reliability of the instrument, and its applicability for use with fully qualified clinicians. Further research is needed to determine whether responses vary as a consequence of professional training background, and therefore whether discipline-specific versions of the instrument should be developed. The current version was developed in Australia, utilizing expert consultation with experts from the United Kingdom and Europe.

## Conclusion

There is a clearly identified need for training and education for nursing staff regarding the metabolic health of mental health service users. The M-BACK tool is a valid and reliable instrument to measure the effectiveness of education and training to improve the attitudes, confidence, and knowledge of mental health nursing staff in relation to metabolic health and changes in perceptions of barriers to delivery of metabolic health care. Mental health services need to incorporate specific training on metabolic care for service users, and the M-BACK questionnaire will be a useful tool in future studies of training outcomes in mental health clinicians.

## Ethics Approval and Consent to Participate

Written and informed consent was obtained from all research participants. Ethical approval was granted for the study by University of Technology Sydney Research Committee (HREC 2013000749).

## Availability of Data and Material

The datasets used and/or analyzed during the current study available from the corresponding author on reasonable request.

## Ethics Statement

Ethical approval was granted for the study by University of Technology Sydney Research Committee (HREC 2013000749).

## Author Contributions

AW was the study coordinator. He led recruitment, data collection, and data analysis. He drafted the conceptual framework and the manuscript. SR was involved in the conception and design of the study and took the lead in supporting AW revising the manuscript. PW assisted the study coordinator with study design and analysis. PW was also involved in revising the conceptual framework. JP assisted the study coordinator with the recruitment, data collection, and manuscript review. ED-W revised the conceptual framework and the manuscript. JS-P assisted with design and conception of the study. She was involved in revising the conceptual framework and manuscript.

## Conflict of Interest Statement

The authors declare that the research was conducted in the absence of any commercial or financial relationships that could be construed as a potential conflict of interest.

## Supplementary Material

Image S1 The M-BACK questionnaire.

Image S2 Scoring for the M-BACK questionnaire.

## Abbreviations

M-BACK, metabolic barriers, attitudes, confidence, and knowledge; SMI, severe mental illness; MetS, metabolic syndrome; ICC, intraclass correlations.
