# Supplementary material for: The Validity and Reliability Characteristics of the M-BACK Questionnaire to Assess the Barriers, Attitudes, Confidence, and Knowledge of Mental Health Staff Regarding Metabolic Health of Mental Health Service Users
Source: Front Public Health. 2017 Dec 11;5:321. doi: 10.3389/fpubh.2017.00321 (PMC5732257; doi:10.3389/fpubh.2017.00321)
Supplement: Image S1 — The M-BACK questionnaire. [file Image_1.PDF]

Reference code_______________ Date_________

The M-BACK Questionnaire

1. My workload prevents me doing any health promotion activities with consumers.

1	2	3	4	5
Strongly Disagree	Disagree	Neutral	Agree	Strongly Agree

2. Consumers with a severe mental illness are not interested in improving their physical health.

1	2	3	4	5
Strongly Disagree	Disagree	Neutral	Agree	Strongly Agree

3. Informing clients about the possible effects medications may have on their physical health will increase non-adherence.

1	2	3	4	5
Strongly Disagree	Disagree	Neutral	Agree	Strongly Agree

4. Screening for metabolic syndrome and physical health interventions are pointless as poor physical health outcomes are unavoidable.

1	2	3	4	5
Strongly Disagree	Disagree	Neutral	Agree	Strongly Agree

5. Metabolic health screening is an important part of my role as a mental health clinician.

1	2	3	4	5
Strongly Disagree	Disagree	Neutral	Agree	Strongly Agree

6. Giving smoking cessation advice is an important part of my role as a mental health clinician.

1	2	3	4	5
Strongly Disagree	Disagree	Neutral	Agree	Strongly Agree

7. Encouraging consumers to increase their level of physical activity is an important part of my role as a mental health clinician.

1	2	3	4	5
Strongly Disagree	Disagree	Neutral	Agree	Strongly Agree

8. Discussing nutritional intake is an important part of my role as a mental health clinician.

1	2	3	4	5
Strongly Disagree	Disagree	Neutral	Agree	Strongly Agree

9. I am confident in my ability to screen for metabolic syndrome.

1	2	3	4	5
Strongly Disagree	Disagree	Neutral	Agree	Strongly Agree

10. I am confident in providing smoking cessation advice to consumers.

1	2	3	4	5
Strongly Disagree	Disagree	Neutral	Agree	Strongly Agree

11. I am confident in prescribing exercise interventions to prevent / treat metabolic syndrome.

1	2	3	4	5
Strongly Disagree	Disagree	Neutral	Agree	Strongly Agree

12. I am confident in using dietary interventions to prevent / treat metabolic syndrome in consumers.

1	2	3	4	5
Strongly Disagree	Disagree	Neutral	Agree	Strongly Agree

13. I have a good knowledge of metabolic syndrome.

1	2	3	4	5
Strongly Disagree	Disagree	Neutral	Agree	Strongly Agree

14. I understand how to screen for metabolic syndrome.

1	2	3	4	5
Strongly Disagree	Disagree	Neutral	Agree	Strongly Agree

15. I understand how to read pathology reports for lipids and glucose results.

1	2	3	4	5
Strongly Disagree	Disagree	Neutral	Agree	Strongly Agree

16. I understand the metabolic side-effect profiles of different neuroleptic medication.

1	2	3	4	5
Strongly Disagree	Disagree	Neutral	Agree	Strongly Agree
