# Supplementary material for: The Validity and Reliability Characteristics of the M-BACK Questionnaire to Assess the Barriers, Attitudes, Confidence, and Knowledge of Mental Health Staff Regarding Metabolic Health of Mental Health Service Users
Source: Front Public Health. 2017 Dec 11;5:321. doi: 10.3389/fpubh.2017.00321 (PMC5732257; doi:10.3389/fpubh.2017.00321)
Supplement: Image S2 — Scoring for the M-BACK questionnaire. [file Image_2.PDF]

Scoring for BACK questionnaire

Attitudes, Confidence and Knowledge (last 12 question) were scored as

Strongly Disagree	1
Disagree	2
Neutral	3
Agree	4
Strongly Agree	5

Scoring was reversed for the Barrier (first 4 questions) questions as they were negatively posed

Strongly Disagree	5
Disagree	4
Neutral	3
Agree	2
Strongly Agree	1

Scoring for each component of BACK (Barriers, Attitudes, Confidence, Knowledge) is out of 20 (with a minimum score of 4)

Total score is out of 80 (with a minimum score of 16)
